# Zeolitic Imidazolate Framework‐8 in Piezo‐Assisted Mechanochemical Oxidation Reactions

**DOI:** 10.1002/open.70265

**Published:** 2026-07-17

**Authors:** Shadi Asgari, Ghodsi Mohammadi Ziarani, Aylar Naderahmadian, Alireza Badiei, Senem Akkoc, Mehran Feizi‐Dehnayebi

**Affiliations:** ^1^ Department of Organic Chemistry Faculty of Chemistry Alzahra University Tehran Iran; ^2^ School of Chemistry College of Science University of Tehran Tehran Iran; ^3^ Department of Basic Pharmaceutical Sciences Faculty of Pharmacy Suleyman Demirel University Isparta Türkiye; ^4^ Faculty of Engineering and Natural Sciences Bahcesehir University Istanbul Türkiye

**Keywords:** ball milling, mechanochemical oxidation reactions, piezocatalyst, zeolitic imidazolate framework‐8

## Abstract

Piezocatalysis, in combination with mechanochemical reactions, can create a respectable revolution in organic synthesis by enhancing product yield. In this study, ball milling merging with the synthesized zeolitic imidazolate framework‐8 (ZIF‐8), possessing a specific surface area of 1097 m^2^/g, was explored to catalyze the oxidative mechanochemical solventless homocoupling reaction of thiols (R‐SH) to disulfides (R‐S–S‐R) at 450 rpm within 120 min, as well as the oxidative mechanochemical solvent‐assisted reaction of toluene to phenol at 450 rpm within 180 min. 1‐butanethiol and 4‐aminothiophenol, as the models of thiol compounds, were oxidized to 1,2‐dibutyldisulfane and 4,4′‐disulfanediyldianiline, respectively, with the yields of 40% and 64%. The production of disulfides was monitored by ^1^H‐NMR, ^13^C‐NMR, and gas chromatography–mass spectrometry. Gas chromatography–flame ionization detection qualitatively and quantitatively explored the phenol production, as the phenol concentration was determined to be 5.550 mmol/L at a pH of 5 and 5.533 mmol/L under the condition of no adjusted pH (the initial pH of ≈3). In the toluene oxidation, O_2_^•−^ species were detected as the main species, through radical trapping experiments. The ZIF‐8 particles could be reused after three cycles with no significant loss in the yield of thiol oxidation product, as further confirmed by their recorded Fourier transform infrared spectrum, powder X‐ray diffraction pattern, and field emission scanning electron microscopy images.

## Graphical abstract

Merging piezocatalysis and mechanochemistry as a novel method in organic synthesis. Mechanochemical reactions of thiols to disulfides and toluene to phenol. Ball milling‐driven oxidative reactions over zeolitic imidazolate framework‐8 piezocatalyst.

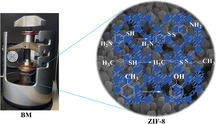

## 1 Introduction

A novel method in organic synthesis is merging piezocatalysis and mechanochemistry, using mechanical energy sources, such as ball milling (BM) or ultrasound (US), to drive the piezocatalysts [1, 2, 3, 4, 5, 6, 7]. The weakening of US waves by increasing the distance from the vibrating surface restricts their application in large‐scale industries [8]. Thus, BM is preferred as a simple technique used in environmentally benign and nonthermal solid‐state reactions [9, 10, 11]. The use of solvent, whether minor or not, is another advantage of the BM technique [12, 13, 14]. A ball mill can generate a huge instant mechanical energy at local positions of piezocatalysts. It leads to the polarization of the piezocatalyst, creating surface defects and crystal lattice distortion, resulting in a substantial increase in catalytic activity [15]. In organic synthesis, BM‐based solid‐state reactions are carried out when the starting materials are insoluble or partially soluble, or when the desired products are difficult or even impossible to yield via liquid‐phase reactions [16, 17, 18, 19, 20]. A piezocatalysis‐mediated mechanochemical organic synthesis is preferred to a traditional mechanochemical organic synthesis due to promoting the yield of products [3]. For example, Schumacher et al. [21] used tet‐BaTiO_3_ nanoparticles (500 nm, 20 wt%) in electric‐assisted BM‐induced copper‐catalyzed atom transfer radical cyclization (ATRC) under the conditions of argon atmosphere, 25 Hz, 90 min, and zirconia balls, to obtain 97% of the ATRC product. Only 5%–6% of the ATRC product was generated when the nonpiezoelectric materials, TiO_2_ (anatase) and Al_2_O_3_ (gamma), were used.

Metal–organic frameworks (MOFs) owe their piezoelectric properties to their crystalline, porous, and customizable structures [22, 23]. However, the piezoelectric behavior of MOFs is rarely reported. The piezoferroelectricity of zeolite imidazolate frameworks (ZIFs) has been lately reported [24]. Because of its high specific surface area, high nitrogen content, open pore structure, and high thermal and chemical stability, ZIF‐8 has a high potential for catalytic applications [25, 26, 27, 28, 29, 30].

Considering the piezoelectricity of ZIF‐8 under BM, the driven ZIF‐8 can promote the mechano‐oxidation reactions under mild and solvent‐free conditions, short reaction times, and operational simplicity. The production of disulfides (R‐S–S‐R) from thiols (R‐S‐H) is an example of the piezocatalytic oxidation reactions, as reported by Wang et al. [31], who used BaTiO_3_ (<3 µm) as the piezocatalyst. The oxidation of 4‐methoxybenzenethiol, as a model of substrate, was performed with a yield of > 99% in a polyethylene (PE) milling jar equipped with stainless steel (SS) balls (4 mm) under air within 6 min. Another example is toluene transformation to phenol, as reported by Song et al. [15] over barium strontium sulfate (Ba_0.75_Sr_0.25_SO_4_, hexagonal crystal structure, 1 − 3 μm) driven by BM. The highest yield of phenol production was obtained at 55.6% when 0.05 g of the piezocatalyst was used for 3 h. Under a pH of 5, the yield of phenol reached 59.4%. A partial conversion of phenol to benzoquinone was observed in more than 3 h.

In this study, ZIF‐8, a recent MOF‐based piezocatalyst, was used in the piezocatalytic mechanochemical oxidation reactions, including the production of phenol from toluene and the production of 1,2‐dibutyldisulfane and 4,4′‐disulfanediyldianiline from the homocoupling reaction of 1‐butanethiol and 4‐aminothiophenol, respectively, under BM.

## 2 Experimental Section

Chemicals, apparatus, and synthesis of ZIF‐8 are provided in the Supporting Information. Figure S1 shows the synthesis of ZIF‐8 in an aqueous solution at room temperature.

### 2.1 Synthesis of 1,2‐Dibutyldisulfane by Oxidation of 1‐Butanethiol

A SS jar (15 mL) was charged with 1‐butanethiol (0.5 mL), ZIF‐8 (0.09 g), and five 5 mm SS balls and sealed. The mechanochemical reaction was carried out for 120 min (every 15 min, with a 5‐minute interval) at 450 rpm in a ball mill, under an initially sealed air atmosphere. The yellowish oily product was finally obtained after washing the mixture with dichloromethane, centrifuging to remove the ZIF‐8 particles, and concentrating by a rotary evaporator. Yield: 40%.

### 2.2 Synthesis of 4,4′‐Disulfanediyldianiline by Oxidation of 4‐Aminothiophenol

The yellow–orange solid product was obtained through the same procedure and conditions as 1,2‐dibutyldisulfane, with one difference that the SS jar was charged with 4‐aminothiophenol (0.5 g) instead of 1‐butanethiol (0.5 mL). Yield: 64%. Melting point: 73–75 °C.

### 2.3 Synthesis of Phenol by Oxidation of Toluene

The SS jar (15 mL) was charged with a suspension of ZIF‐8 particles (0.05 g) in acetonitrile (800 µL) and toluene (100 µL), along with five 5 mm SS balls, and sealed. The suspension's initial pH was about 3. The mechanochemical reaction was carried out for 180 min (every 30 min, with a 5‐minute interval) at 450 rpm in a ball mill, under an initially sealed air atmosphere. The reaction mixture was washed with dried methanol (three times, each time with 10 mL of methanol) and centrifuged to remove the ZIF‐8 particles from the phenol solution. The phenol solution was then collected for gas chromatography–flame ionization detection (GC–FID) to qualitatively and quantitatively determine the phenol.

Because of phenol's weak acidity, the effect of pH on the reaction was explored. According to the most related study [15], a pH of 5 was reported as the optimum. Thus, we explored the reaction at this pH. At the adjusted pH of 5, the synthesis procedure was the same; only a NaOH solution (1 M, ≈400 µL) was added to the suspension to change the initial pH from 3 to 5. A Universal Litmus Test Paper pH 0–14 was used to measure pH.

All of the oxidation reactions were also performed in the absence of the ZIF‐8 piezocatalyst for comparison.

In all of the reaction procedures, the ball‐milling jar was in a closed state, as no further air or oxygen could be purged into the jar to increase the molar amount of oxygen.

## 3 Results and Discussion

**FIGURE 1:**
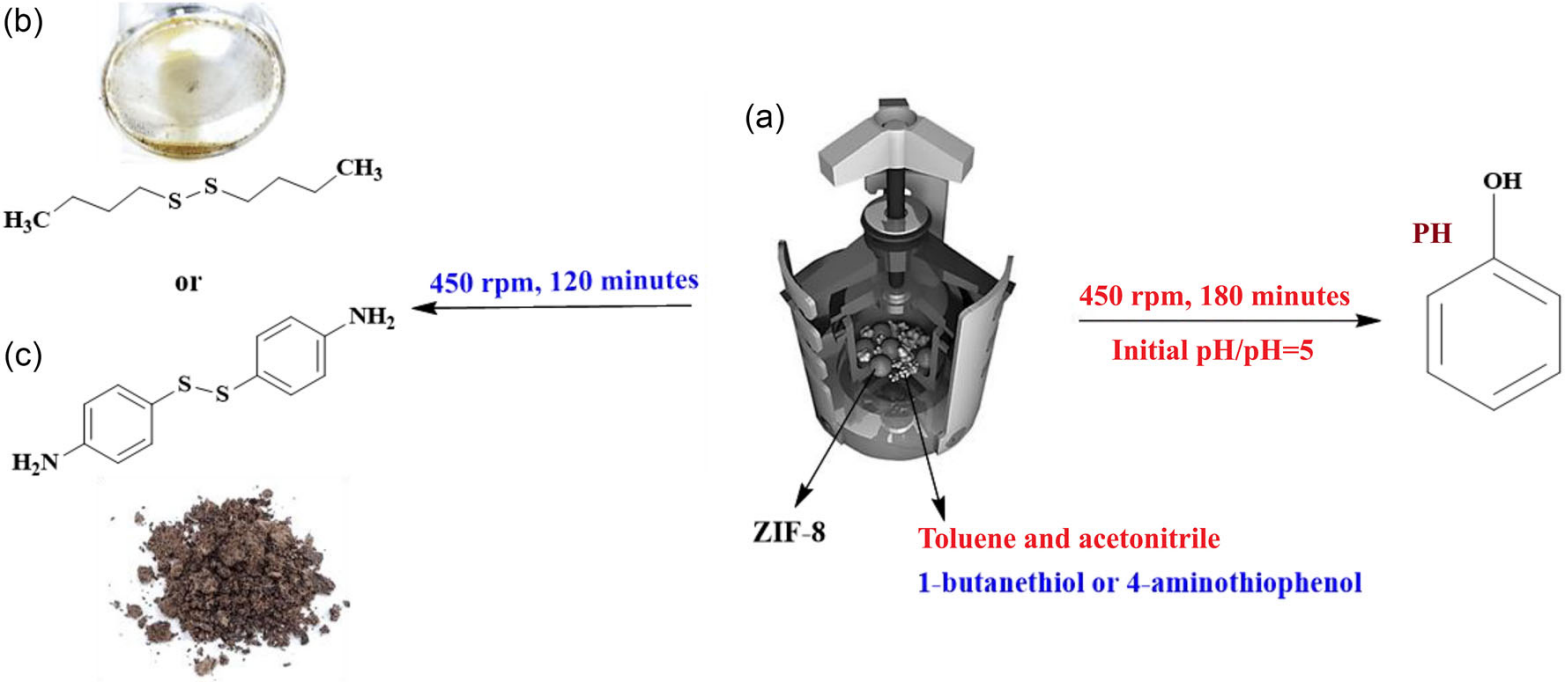
An illustration of the synthetic procedures of (a) phenol, (b) 1,2‐dibutyldisulfane, and (c) 4,4′‐disulfanediyldianiline over ZIF‐8 driven by BM.

In the presence of ZIF‐8, 1‐butanethiol was oxidized to 1,2‐dibutyldisulfane, and 4‐aminothiophenol was oxidized to 4,4′‐disulfanediyldianiline under BM at 450 rpm within 120 min. Toluene was oxidized to phenol under both the initial pH of ≈3 and a pH of 5 at 450 rpm and 180 min. The experimental procedures are shown in Figure 1. For the oxidation reactions in the absence of ZIF‐8, the yield of the products decreased significantly. 1,2‐dibutyldisulfane and 4,4′‐disulfanediyldianiline were formed in low yields of ≈4%–5%, as it was difficult to collect them. In the oxidation of toluene, no phenol was detected by GC–FID.

### 3.1 Characterization of Zeolitic Imidazolate Framework‐8

The Fourier transform infrared (FT‐IR) spectrum, powder X‐ray diffraction (PXRD) pattern, ultraviolet–visible diffuse reflectance spectroscopy spectrum, and N_2_ adsorption–desorption isotherm of ZIF‐8 are shown in Figure S2. Figure S2a indicates the FT‐IR spectrum of ZIF‐8. The peaks located at 3131 and 2928 cm^−1^ are attributed to C–H stretching, which is present in the imidazole ring and in the linker's methyl group [32]. The C=N stretching appeared at 1583 cm^−1^, while the entire ring stretching corresponds to a peak located at 1394 cm^−1^. The peaks located in the range from 900 to 1305 cm^−1^ are related to the in‐plane bending of the ring, and the peaks at 754 and 690 cm^−1^ are associated with aromatic sp^2^ C–H bending [33]. As shown in Figure S2b, the intense reflections were observed at 2*θ* of 7.35°, 10.35°, 12.75°, 14.7°, 16.35°, and 18°, which, respectively, correspond to (011), (002), (112), (022), (013), and (222) planes, confirming a high degree of crystallinity in the synthesized ZIF‐8 [34]. In Figure S2c, the peaks observed at 240 nm and 260 nm are related to the free ionic species, and a broad peak around 380 nm is related to the tetrahedrally coordinated Zn ions [35]. Figure S2d indicates the N_2_ adsorption–desorption isotherm of ZIF‐8, showing a microporous structure (type‐I) as the steepness of the nitrogen uptake occurred at low P/P_0_ [36]. The specific surface area (*S*_BET_), pore size diameter (d_*n*_), and pore size volume (V) of ZIF‐8 were measured at 1097 m^2^/g, 0.129 cm^3^/g, and 2.15 nm, respectively. The pore size distribution determined by BJH is shown in Figure S3.

**FIGURE 2:**
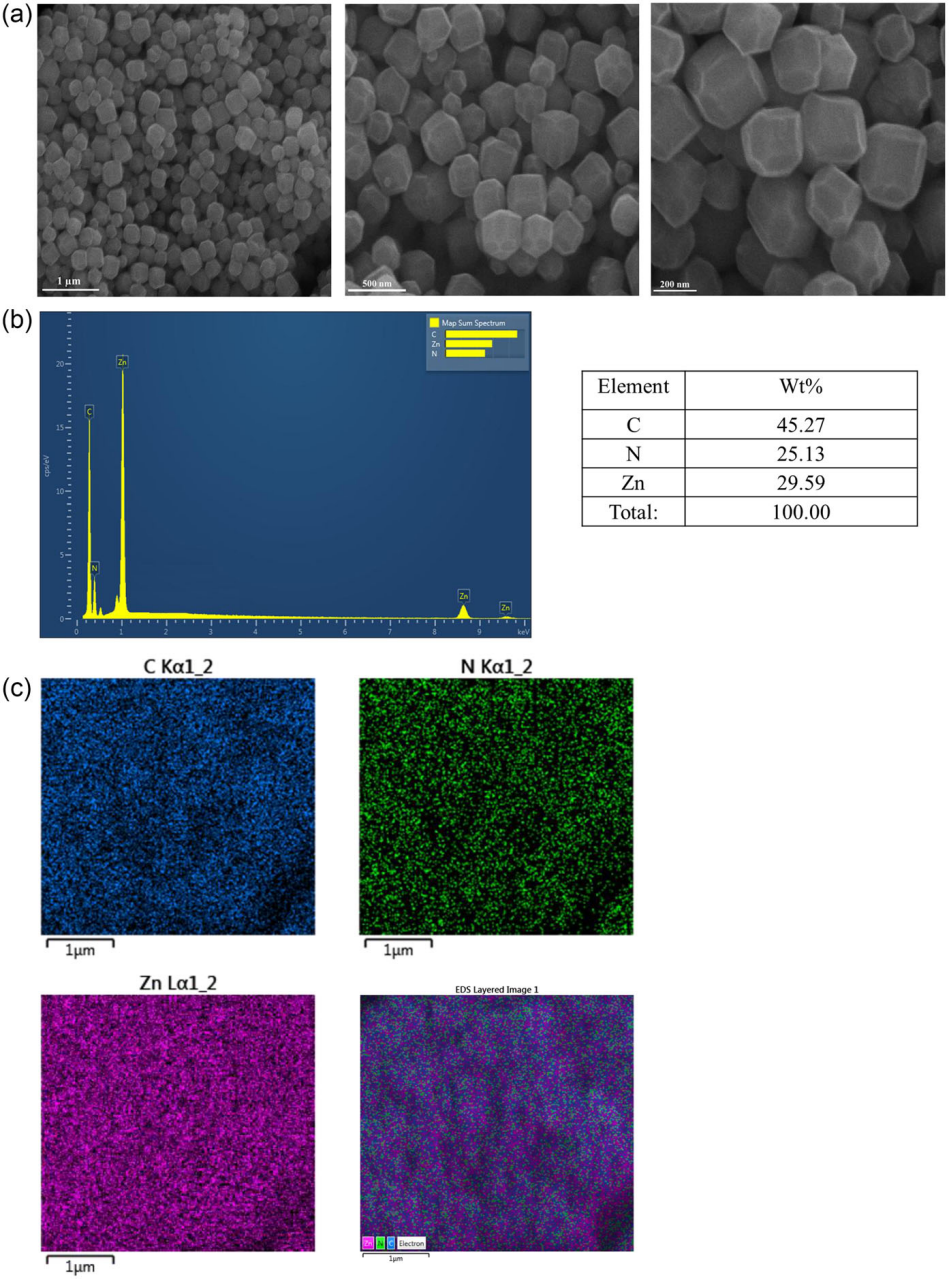
(a) Morphology, (b) elemental composition, and (c) dot‐mapping images of the synthesized ZIF‐8.

The morphology and composition of the ZIF‐8 particles were evaluated by field emission scanning electron microscopy (FE‐SEM)/energy‐dispersive X‐ray analysis/elemental mapping analysis, as shown in Figure 2. FE‐SEM images showed nanoparticles with sharp hexagonal facets. The average particle size was determined to be 324 ± 74 nm, calculated with ImageJ. The ZIF‐8 particles indicated the characteristic elements of C, N, and Zn, distributed homogenously, as detected in the dot‐mapping images (Figure 2c).

### 3.2 Characterization of 1,2‐Dibutyldisulfane and 4,4′‐Disulfanediyldianiline

#### 3.2.1 Nuclear Magnetic Resonance Spectroscopy

**FIGURE 3:**
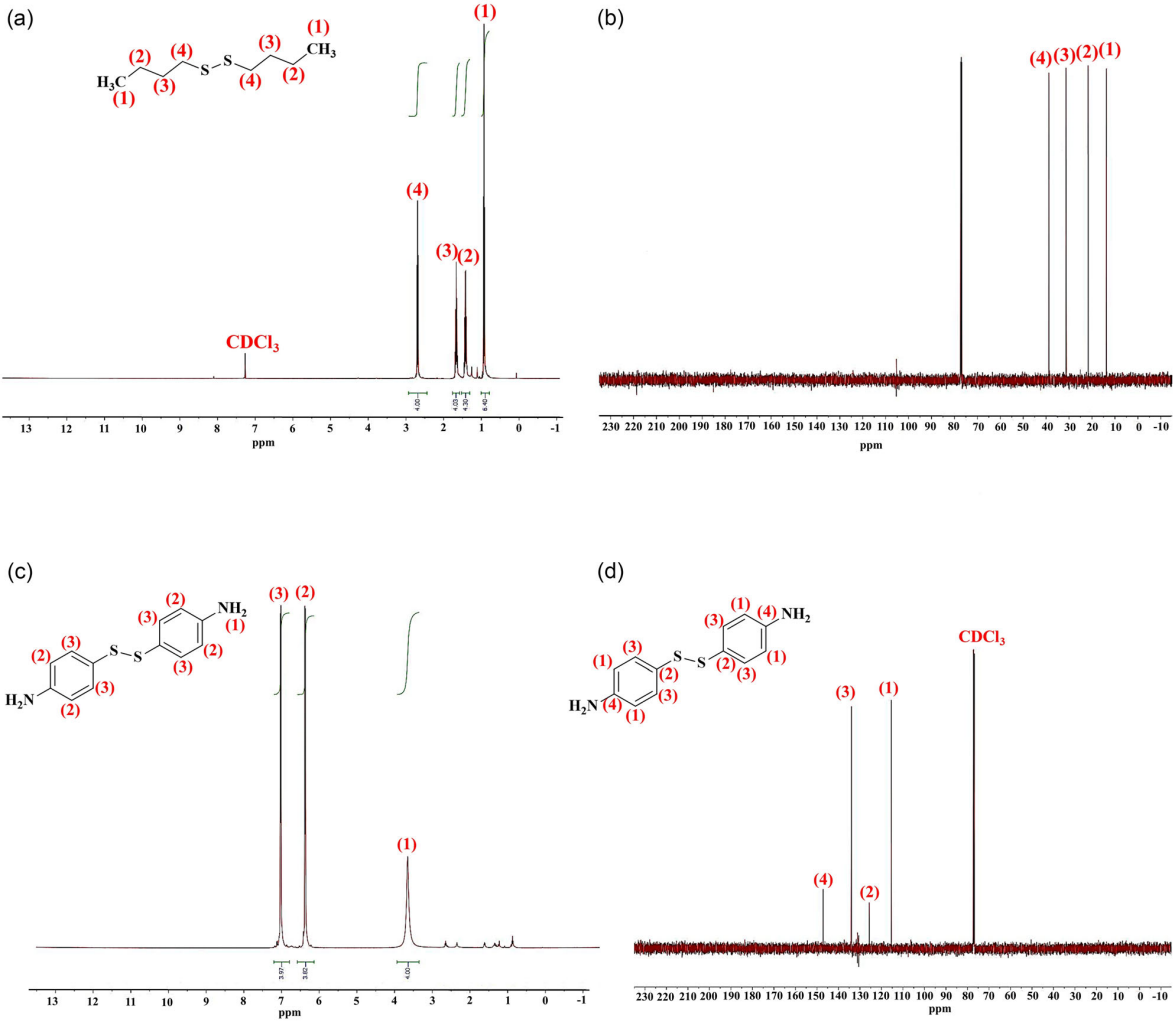
(a) ^1^H‐NMR and (b) ^13^C‐NMR spectra of 1,2‐dibutyldisulfane. (c) ^1^H‐NMR and (d) ^13^C‐NMR spectra of 4,4′‐disulfanediyldianiline.

The successful oxidation of 1‐butanethiol and 4‐aminothiophenol was proved by recording the ^1^H‐NMR and ^13^C‐NMR spectra of their oxidation products in CDCl_3_. The ^1^H‐NMR and ^13^C‐NMR spectra of 1,2‐dibutyldisulfane are shown in Figure 3a,b, respectively. The distinctive hydrogen chemical shifts were observed at 2.71 (4H), 1.66 (4H), 1.41 (4H), and 0.94 ppm (6H), and the carbon chemical shifts were observed at 39.08, 31.36, 21.67, and 13.95. Figure 3c,d is attributed to the ^1^H‐NMR and ^13^C‐NMR spectra of 4,4′‐disulfanediyldianiline, respectively. The characteristic hydrogen chemical shifts were observed at 7.27 (4H), 6.63 (4H), and 3.81 (4H), and the carbon chemical shifts were detected at 147.01, 134.09, 125.89, and 115.34. The magnified ^1^H‐NMR spectra, in which the peak splitting is observed, are indicated in the Supporting Information (Figure S4). The full original nuclear magnetic resonance (NMR) spectra (MestReNova documents) are provided in the Supporting Information.

#### 3.2.2 Gas Chromatography–Mass Spectrometry

**FIGURE 4:**
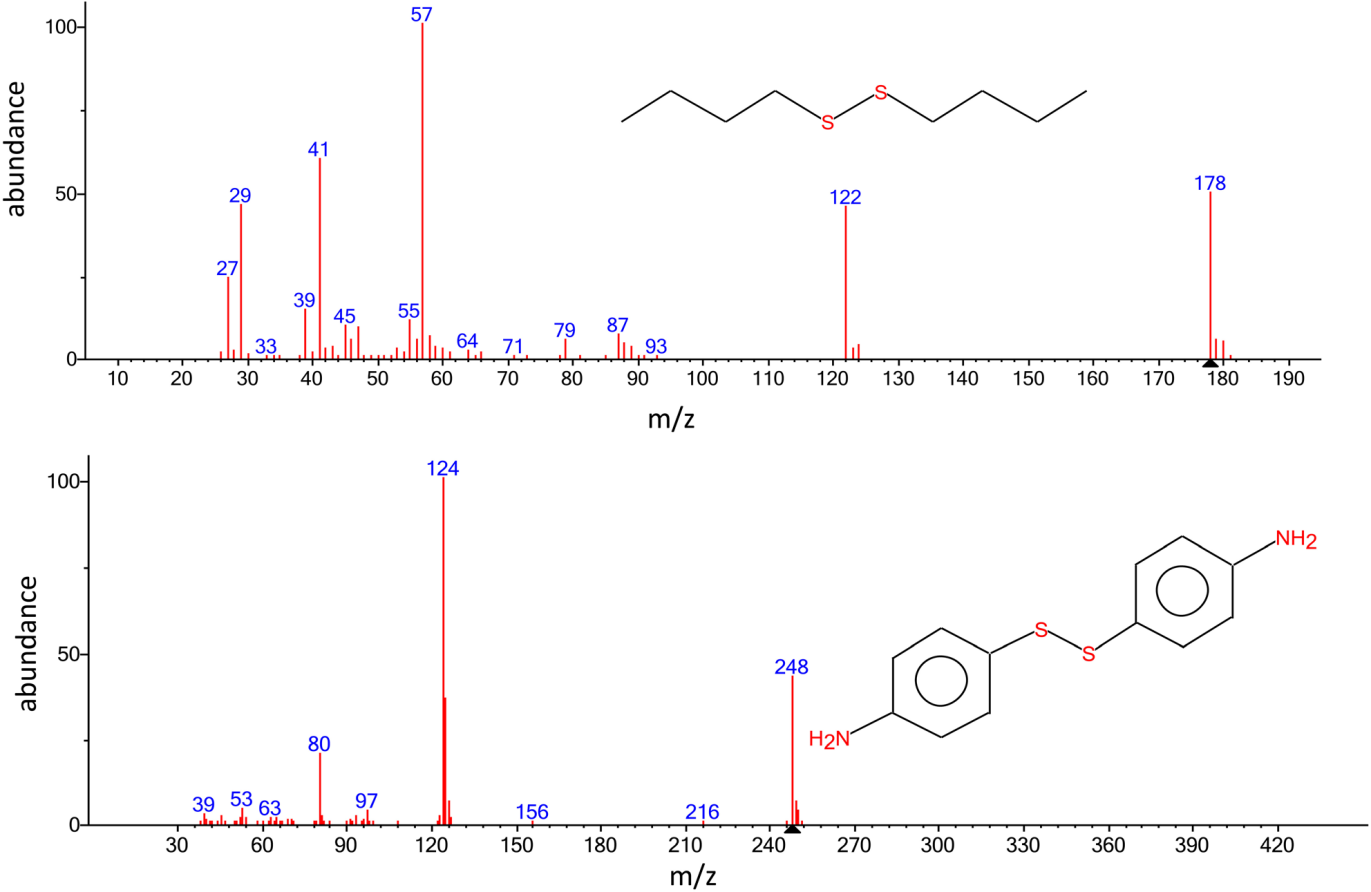
The *m*/*z* data obtained from GC–MS for 1,2‐dibutyldisulfane and 4,4^′^‐disulfanediyldianiline.

Gas chromatography–mass spectrometry (GC–MS) was applied to monitor the accomplishment of the oxidation reactions. The identified peaks appeared at 57, 122, and 178 *m*/*z*, indicating the generation of 1,2‐dibutyldisulfane, and the characteristic peaks located at 80, 124, and 248 *m*/*z* are a witness to the successful generation of 4,4′‐disulfanediyldianiline (Figure 4). GC–MS chromatograms are provided in the Supporting Information (Figure S5). The retention times were detected at ≈6.5 and 13.7 min for 1,2‐dibutyldisulfane and 4,4′‐disulfanediyldianiline, respectively.

### 3.3 Evaluation of Phenol

#### 3.3.1 Evaluation of Phenol Production by Gas Chromatography–Flame Ionization Detector (GC–FID)

The standard solutions of phenol were prepared at 2, 3, 5, and 6 mmol/L in methanol, and the produced phenol concentration was calculated from the standard curve. After GC detection, the *X*‐axis was set as the phenol concentration (mmol/L), and the *Y*‐axis as the GC peak area of phenol (pA*s), as shown in Figure S6. The phenol concentration was calculated via the formula of *y* = 1.2088x + 0.9223. By placing the *Y*‐axis value related to the retention time of ≈2.6, that is for phenol, the *X*‐axis, that is the phenol concentration in the unknown solution, was calculated to be 5.550 mmol/L at a pH of 5 and 5.533 mmol/L at the initial pH of ≈3.

This insignificant difference can be due to the rearrangement of toluene hydrogen peroxide, and intermediate products to produce phenol at the pH of 5 [15, 37]. Indeed, at a higher acidity, more reaction by‐products, such as water, are introduced into the reaction environment, which are not favorable to the forward reaction progress. At a higher basicity, the yield of phenol is expected to decrease because the hydrogen ion concentration decreases significantly under alkaline conditions, so the rearrangement reaction of toluene hydrogen peroxide cannot be performed, and the yield of phenol decreases [15]. The GC–FID chromatograms of the phenol standard solutions, along with the phenol solutions at the pH of 5 and the initial pH, are provided in the Supporting Information (Figure S7).

#### 3.3.2 Explore the Reaction Mechanism

**FIGURE 5:**
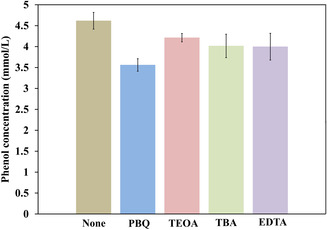
The effect of radical trapping agents on the produced phenol concentration over ZIF‐8.

Considering the decrease in phenol concentration in the presence of radical trapping agents, the reaction mechanism can be clarified. As shown in Figure 5, after adding all trapping agents, the phenol concentration decreased (as determined by GC), indicating that all four species affect the oxidation of toluene. However, a greater influence was observed for capturing the superoxide radicals (O_2_^•−^), indicating the probable attack of the O_2_^•−^ species on the methyl of toluene to generate toluene hydrogen peroxide. Toluene hydrogen peroxide undergoes a rearrangement reaction under acidic conditions (pH of 5) to generate phenol and formaldehyde gas [37]. Hydroxyl radicals (^•^OH) can also be produced in the reaction process as secondary oxidation species to react with toluene to produce phenol [38].

### 3.4 Stability Studies of Zeolitic Imidazolate Framework‐8

**FIGURE 6:**
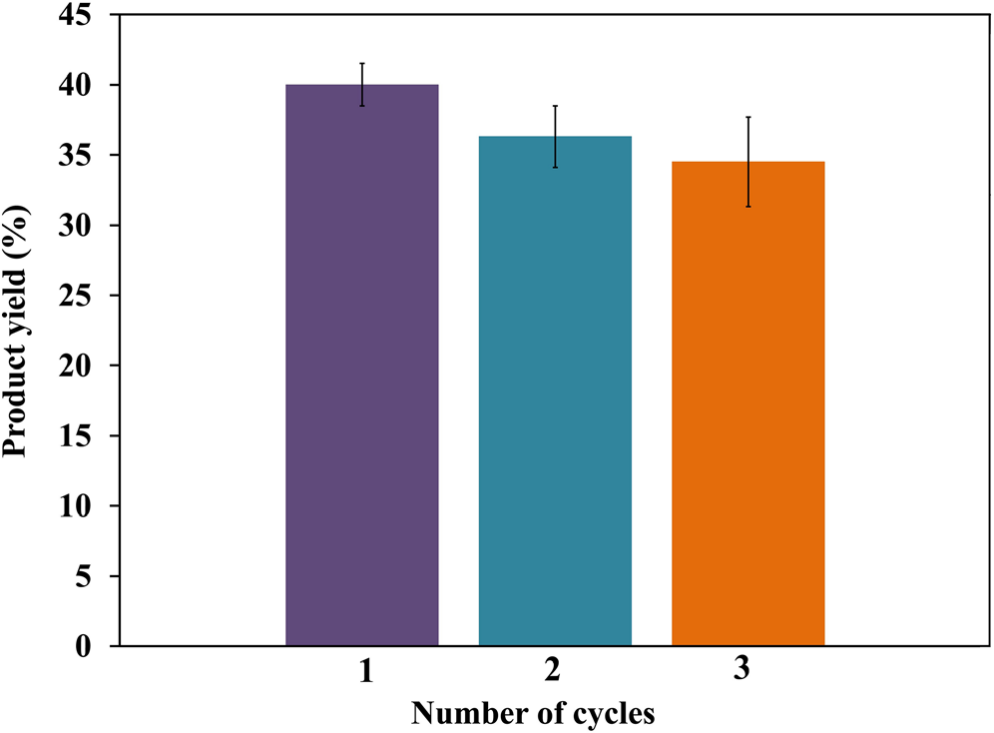
Cycling runs for the oxidation reaction of 1‐butanethiol over ZIF‐8.

The economic capability of a catalyst depends on its stability after several cycles. Thus, the evaluation of the reusability of catalysts is essential [39]. Figure 6 indicates that the product yield for the oxidation of 1‐butanethiol decreased slightly after three cycles using ZIF‐8.

**FIGURE 7:**
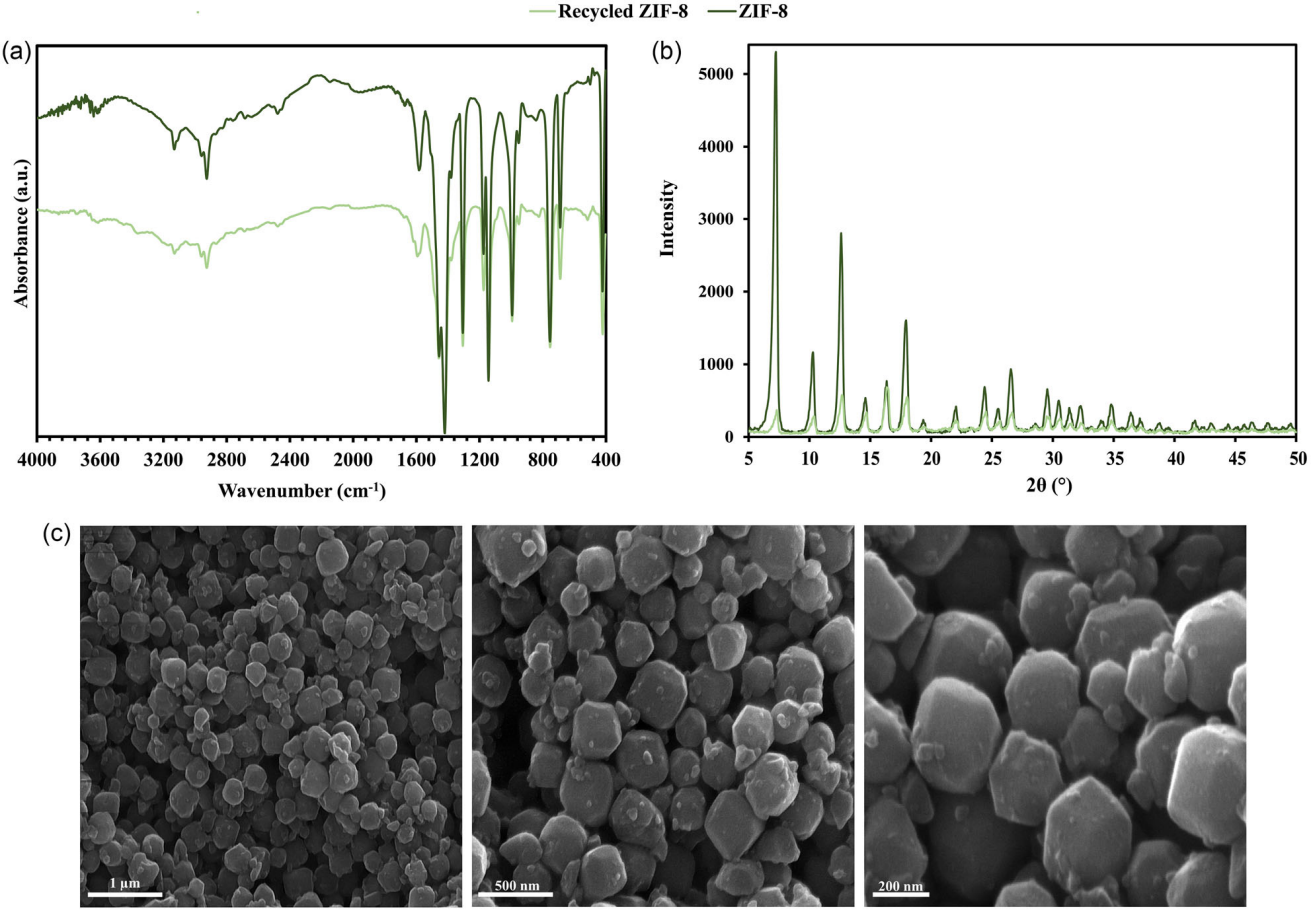
(a) FT‐IR spectrum, (b) PXRD pattern, and (c) FE‐SEM images of the ZIF‐8 particles after three cycles of use in the oxidation reaction of 1‐butanethiol.

The high reusability of the ZIF‐8 particles was further confirmed by recording their FT‐IR spectrum, PXRD pattern, and FE‐SEM images after three cycles of their use in the oxidation reaction of 1‐butanethiol. As shown in Figure 7a, the IR peak positions of the recycled ZIF‐8 were similar to those of ZIF‐8, confirming that it is still active for further reactions. Figure 7b shows that the PXRD pattern of ZIF‐8 is identical before and after recycling, with only a reduction in the diffraction intensity of the recycled ZIF‐8. FE‐SEM images, as shown in Figure 7c, also confirm this claim because only minor degradations were observed for the recycled ZIF‐8, and it maintained its hexagonal shape after three cycles. The elemental composition and dot‐mapping images (Figure S8) of the recycled ZIF‐8 indicated the additional elements of S and O, which can be attributed to the thiol compounds and solvent molecules attached to the surface of ZIF‐8 particles after three cycles of use.

Considering the probable instability of ZIF‐8 under acidic conditions, the PXRD pattern of ZIF‐8 was recorded after completing the oxidation reaction of toluene at the pH of 5. As shown in Figure S9, the structure of ZIF‐8 has changed somewhat when compared with the PXRD pattern belonging to the pristine ZIF‐8, previously shown in Figure 7b. The low phenol concentration obtained at both the initial pH and pH of 5 can explain this observation.

## 4 Conclusions and a Brief Comparison Study

A MOF‐based piezocatalyst, ZIF‐8, with a hexagonal structure, high specific surface area, and an average diameter of ≈324, was easily synthesized in water. BM was used as the mechanical energy source to drive the ZIF‐8 piezocatalyst to catalyze the oxidative homocoupling reactions of 1‐butanethiol to 1,2‐dibutyldisulfane and 4‐aminothiophenol to 4,4′‐disulfanediyldianiline and the oxidation reaction of toluene to phenol. These oxidation reactions were confirmed by ^1^H‐NMR and ^13^C‐NMR analyses. GC–MS monitored the successful oxidation of thiols, and GC–FID explored the phenol production at a pH of 5 and at the initial pH of ≈3. The O_2_^•−^ species were distinguished as the main species in the toluene oxidation. The recycled ZIF‐8 indicated approximately similar FT‐IR spectrum, PXRD pattern, and FE‐SEM images compared to those of the pristine ZIF‐8, confirming its high reusability after three cycles of use.

It is valuable to compare the performance of ZIF‐8 in the oxidation reactions with the performance of other piezocatalysts under approximately similar conditions. In the most relevant study on the oxidation of thiols, Gefei Wang et al. (2022) [31] used the commercially available BaTiO_3_ (200 nm, 0.6–1 µm, and < 3 µm) under an MSK‐SFM‐12 M mixer mill (2 mL PE milling jar equipped with five 4 mm SS balls) at 3800 rpm for 6–15 min. The aerobic oxidation of 1‐butanethiol and 4‐aminothiophenol achieved the product yield of > 99% at 6 min and > 99% at 9 min, respectively, showing more piezocatalytic performance of BaTiO_3_ compared to our synthesized ZIF‐8 particles at a lower time but at a significantly higher BM speed. For the oxidation of toluene, Limin Song et al. (2022) [15] used the synthesized Ba_0.75_Sr_0.25_SO_4_ (BSS, hexagonal crystals, 1 − 3 μm) to catalyze the oxidation of toluene to phenol under BM at 600 rpm for 3 h. BaTiO_3_, SrTiO_3_, and lead zirconate titanate (PZT) were also explored at the same experimental conditions. BSS indicated the best catalytic performance, and the phenol yield reached 31.6% at 0.01 mg and 55.6% at 0.05 mg of BSS catalyst. Through exploring the effect of pH value on the reaction, the optimum reaction condition was determined to be a pH of 5, in which the yield of phenol was 59.4%.

## Author Contributions

**Shadi Asgari:** writing original draft, doing experiments, and data collection**. Ghodsi Mohammadi Ziarani:** project administration‐lead, supervision‐lead, editing‐lead. **Aylar Naderahmadian:** data collection**. Alireza Badiei:** review and editing**. Senem Akkoc:** review and editing. **Mehran Feizi‐Dehnayebi:** review and editing.

## Funding

The authors have nothing to report.

## Consent of Publication

All authors read and approved the final manuscript.

## Conflicts of Interest

The authors declare no conflicts of interest.

## Supporting information

Supplementary Material

## Data Availability

Data will be made available on request to the corresponding author.
