# Supplementary material for: Zeolitic Imidazolate Framework‐8 in Piezo‐Assisted Mechanochemical Oxidation Reactions
Source: ChemistryOpen. 2026 Jul 17;15(8):e70265. doi: 10.1002/open.70265 (PMC13378737; doi:10.1002/open.70265)
Supplement: Supplementary file 1 — Supplementary Material [file OPEN-15-e70265-s001.pdf]

Supporting Information

ZIF-8 in piezo-assisted mechanochemical oxidation reactions

Shadi Asgari[a], Ghodsi Mohammadi Ziarani[a*], Aylar Naderahmadian[b], Alireza Badiei[b*], Senem Akkoc[c,d*], Mehran Feizi-Dehnayebi[c]

[a] Department of Organic Chemistry, Faculty of Chemistry, Alzahra University, Tehran P.O. Box 1993893973, Iran.

[b] School of Chemistry, College of Science, University of Tehran, Tehran, Iran.

[c] Department of Basic Pharmaceutical Sciences, Faculty of Pharmacy, Suleyman Demirel University, Isparta, Türkiye.

[d] Bahcesehir University, Faculty of Engineering and Natural Sciences, Istanbul, 34353, Türkiye.

Corresponding Authors:
[*] *gmohammadi@alzahra.ac.ir*
[*] *abadiei@ut.ac.ir*
[*] *senemakkoc@sdu.edu.tr*

Chemicals and Apparatus

Zinc nitrate hexahydrate (Zn $(NO_3).6H_2O$), 2-Methylimidazole (2-MeIM), methanol, 1-butanethiol, 4-aminothiophenol, dichloromethane, toluene, phenol, acetonitrile, and sodium hydroxide (NaOH) were purchased from Merck. Fourier-transform infrared (FT-IR) spectra of the pristine ZIF-8 and recycled ZIF-8 were recorded on a Bruker Tensor 27 FT-IR spectrometer. The diffuse reflectance ultraviolet–visible (UV–Vis DRS) spectrum of ZIF-8 was collected on a diffuse reflectance spectroscope (UV-2600, SHIMADZU). The powder X-ray diffraction (PXRD) patterns were recorded using a HAOYUAN-DX2700BH X-ray diffractometer at 2θ ranging from 5° to 50°. The surface morphology and elemental composition of the ZIF-8 before and after oxidation reactions were recorded on a field emission scanning electron microscope (FE-SEM) coupled with an energy dispersive X-ray (EDAX) spectroscope (MIRA3, Tescan). The ZIF-8 particles were activated at 110 ºC overnight to then measure their specific surface area, pore size distribution, and pore size volume with an N_2 adsorption-desorption experiment at 77 K on Micromeritics® TriStar II Plus. ^{1}H-NMR and ^{13}C-NMR spectra of 4,4'-disulfanediyldianiline and 1,2-dibutyldisulfane products were collected on an NMR spectroscope (VARIAN-INOVA 500 MHz) in $CDCl_3$. An Agilent 6890N Network Gas Chromatograph was used for qualitative and quantitative detection of phenol under the conditions, including the temperature oven of 60-280 °C, rate of 20 °C/min, inlet temperature of 275 °C, detector temperature of 285 °C, splitless sample way, injection volume of 1 µL, and carrier gas of N_2. An Agilent GC/MS 6890 Plus-5973N MSD was applied to record the GC-MS spectra to confirm the successful oxidation of thiols to disulfides. The GC-MS conditions include the oven temperature of 60-280 °C, heating rate of 20 °C/min, inlet temperature of 275 °C, ion source and interface temperatures of 300 °C, split ratio of 1:50, injector pressure of 1 µL, and carrier gas of He. A planetary ball mill (BM) (NARYA-MPM 2*250H; a 15 mL stainless steel (SS) milling jar charged with five 5 mm SS balls) was used as a mechanical energy source for the piezocatalytic oxidation reactions. The program settings of BM were as follows:

For oxidation of thiols

Set time (min): 15

Rest (min): 5

Speed (rpm): 450

Repeat: 8

For oxidation of toluene:

Set time (min): 30

Rest (min): 5

Speed (rpm): 450

Repeat: 6

As is clear in the above-mentioned program settings, the interval time is not included in the total reaction time. For example, for the oxidation of thiols, the total reaction time was 120 minutes, and the total interval time was 40 minutes; thus, the total consumed time was 160 minutes. Likewise, the total consumed time was 210 minutes for the oxidation of toluene.

Synthesis of Zeolitic Imidazolate Framework-8 (ZIF-8)

The rapid synthesis of ZIF-8 was performed in an aqueous solution and at room temperature, as first reported by *Yichang Pan et al.*[1] The aqueous solution of Zn $(NO_3)_2.6H_2O$ (0.893 g or 3 mmol in 10 mL of deionized water) was added to the aqueous solution of 2-MeIM (0.985 g, or 12 mmol in 90 mL of deionized water) and stirred for 1 hour at room temperature. The product was separated by centrifuge, washed with methanol three times, and dried at 50°C for 12 hours. The dried ZIF-8 was activated overnight at 110 °C for use in the piezocatalytic oxidation reactions.

Explore the mechanism of the phenol production reaction

Four radical trapping agents were added to the suspension of ZIF-8 at the initial pH of ~ 3. P-benzoquinone (PBQ), ethylene diamine tetra-acetic acid (EDTA), tertiary butyl alcohol (TBA), and triethanolamine (TEOA) were used to trap superoxide radicals ($O_2^{\bullet-}$), free holes (h^+), hydroxide radicals ($^{\bullet}OH$), and free electrons (e^-), respectively. These radical trapping agents can capture the corresponding radical species and reduce the yield. Considering the degree of decline of phenol yield, the reaction mechanism is clarified.

Explore the reusability of ZIF-8

The ZIF-8 particles were used in three cycles of the oxidation reaction of 1-butanethiol. In each cycle, the ZIF-8 particles were separated from the reaction mixture by centrifuging, washed with water, and dried to use in the next cycle. The three cycles were followed by the same procedure for the oxidation reactions.

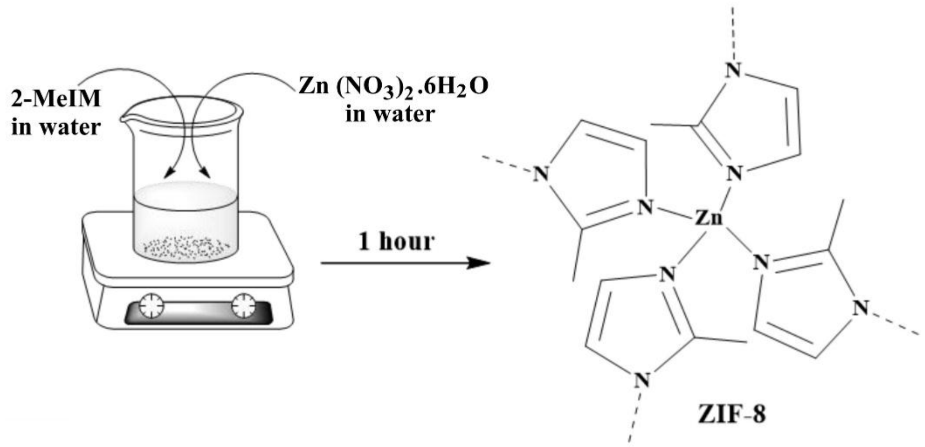

Fig. S1. The synthetic procedure of ZIF-8.

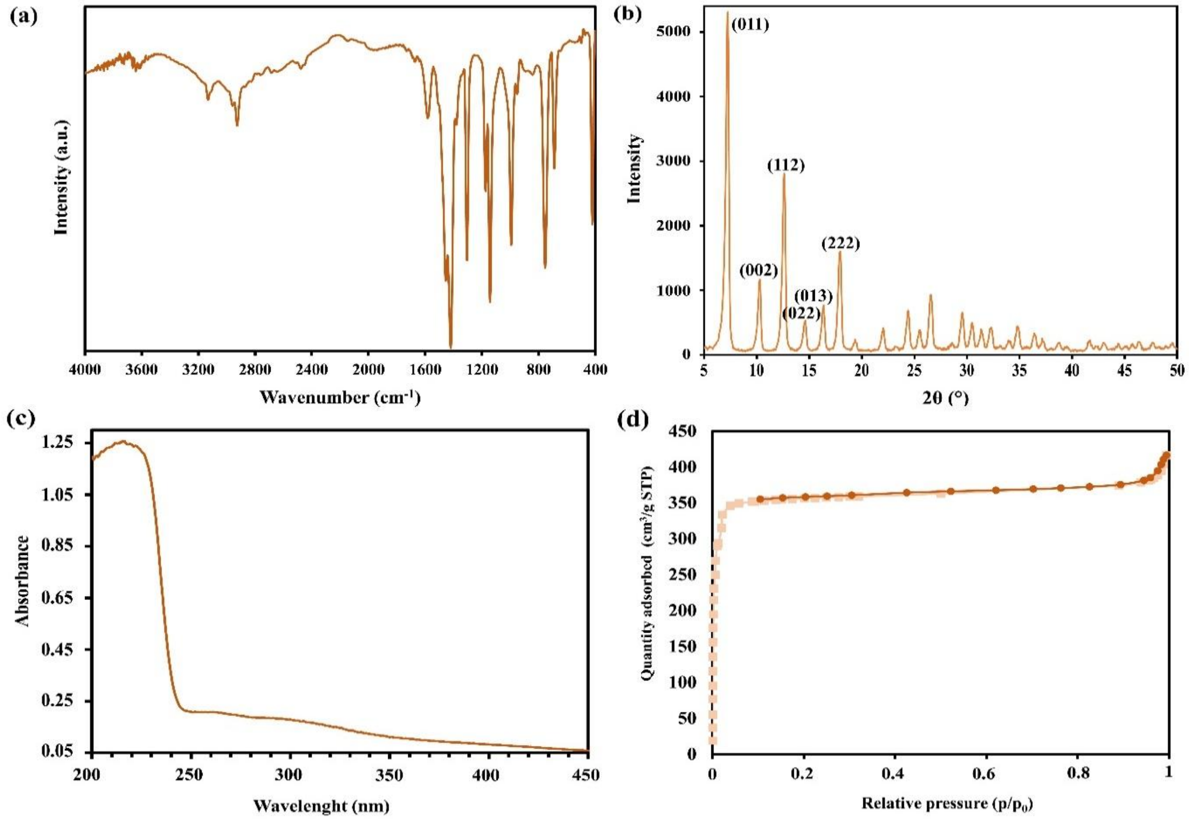

Fig. S2. (a) FT-IR spectrum, (b) PXRD pattern, (c) UV-Vis DRS spectrum, and (d) N_2 adsorption-desorption isotherm of the synthesized ZIF-8.

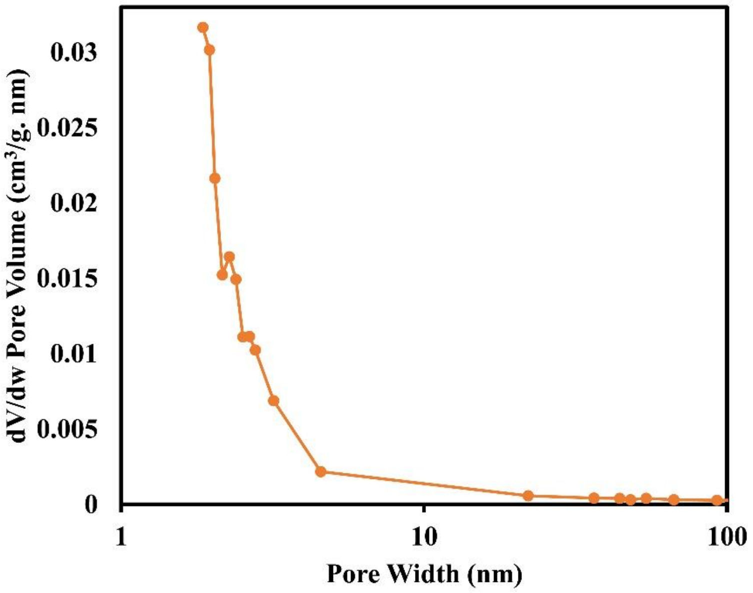

Fig. S3. The pore size distribution of ZIF-8 particles.

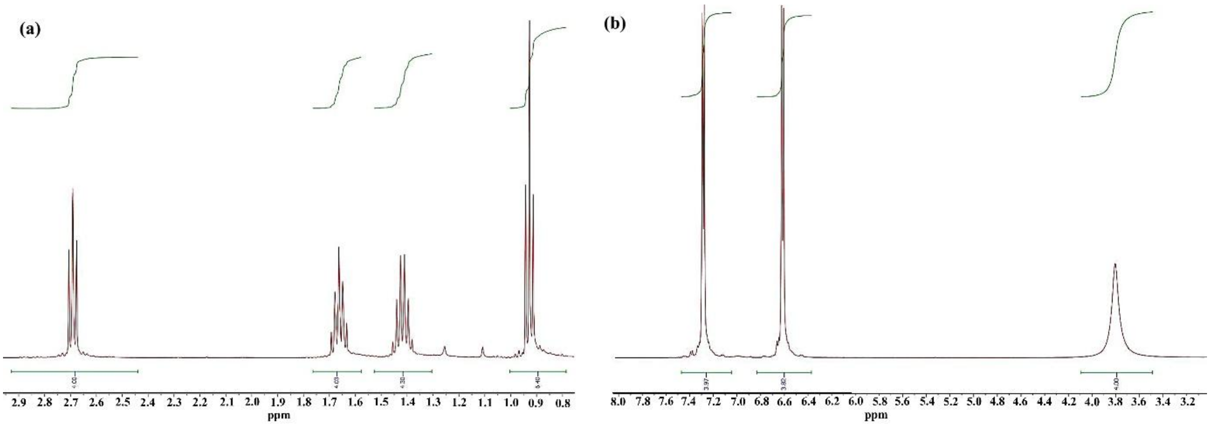

Fig. S4. The magnified ^{1}H-NMR spectra of (a) 1,2-dibutyldisulfane and (b) 4,4'-disulfanediyldianiline.

Original NMR spectra:

1H-NMR of 1,2-dibutyldisulfane.mnova

13C-NMR of 1,2-dibutyldisulfane.mnova

1H-NMR of 4,4'-disulfanediyldianiline.mnova

13C-NMR of 4,4'-disulfanediyldianiline.mnova

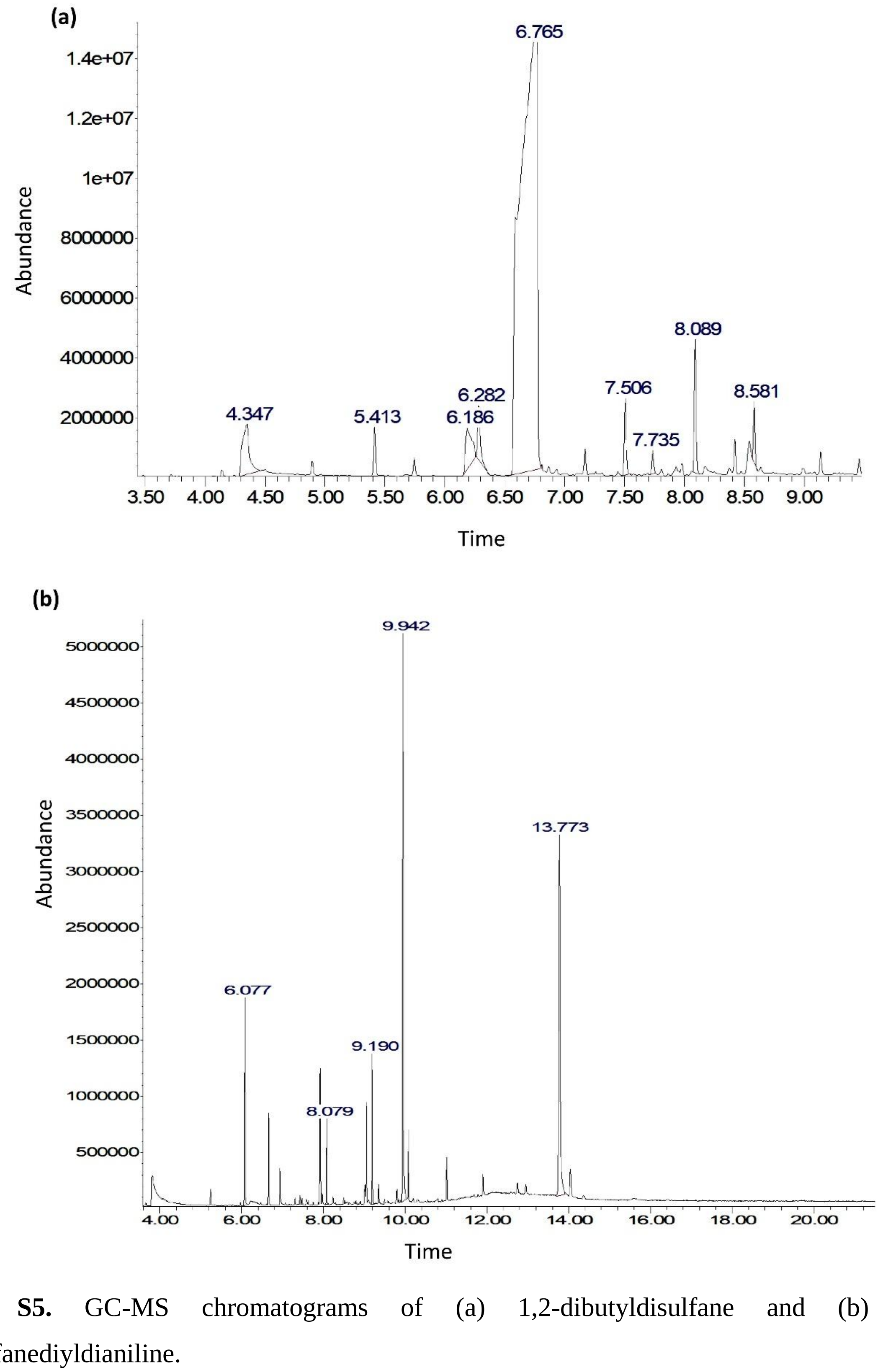

Fig. S5. GC-MS chromatograms of (a) 1,2-dibutyldisulfane and (b) 4,4'-disulfanediyldianiline.

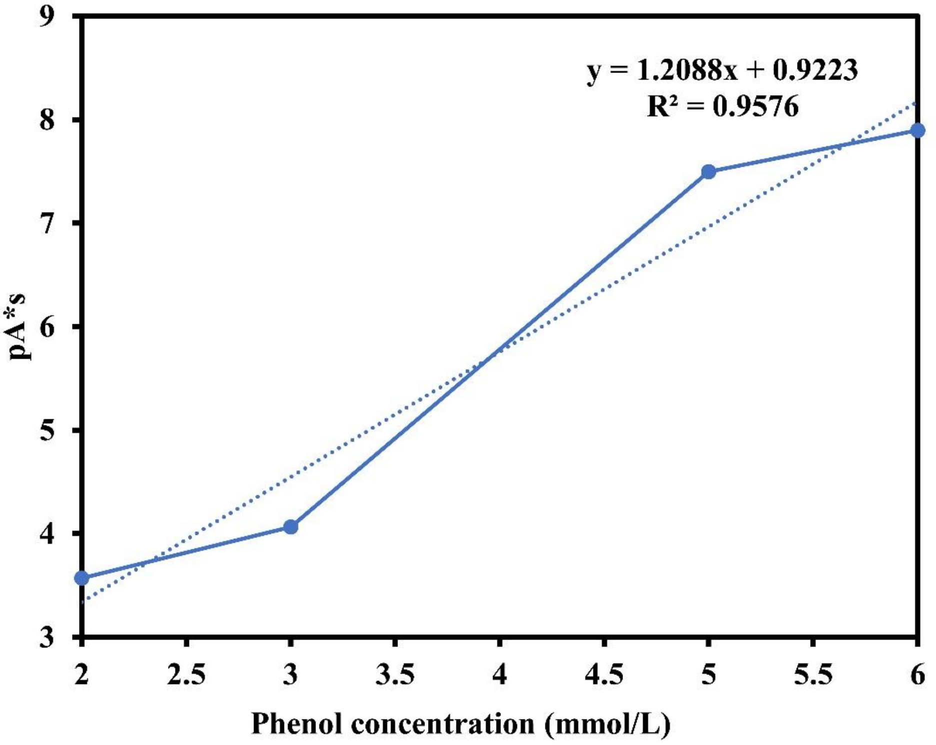

Fig. S6. The phenol standard calibration curve.

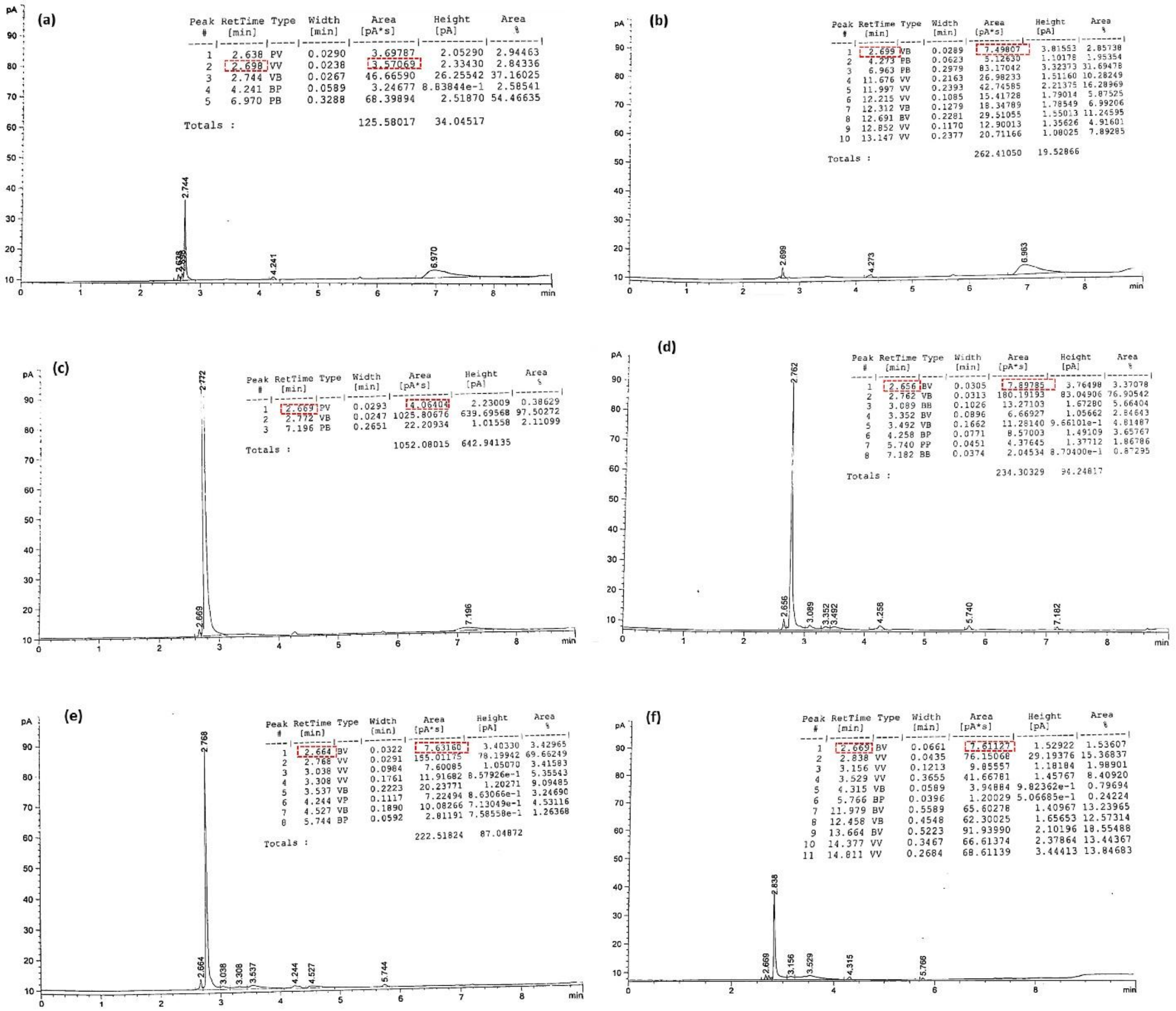

Fig. S7. The GC-FID chromatograms of the phenol standard solutions of (a) 2 mmol/L, (b) 5 mmol/L, (c) 3 mmol/L, and (d) 6 mmol/L, and the phenol solutions at (e) the pH of 5 and (f) the initial pH.

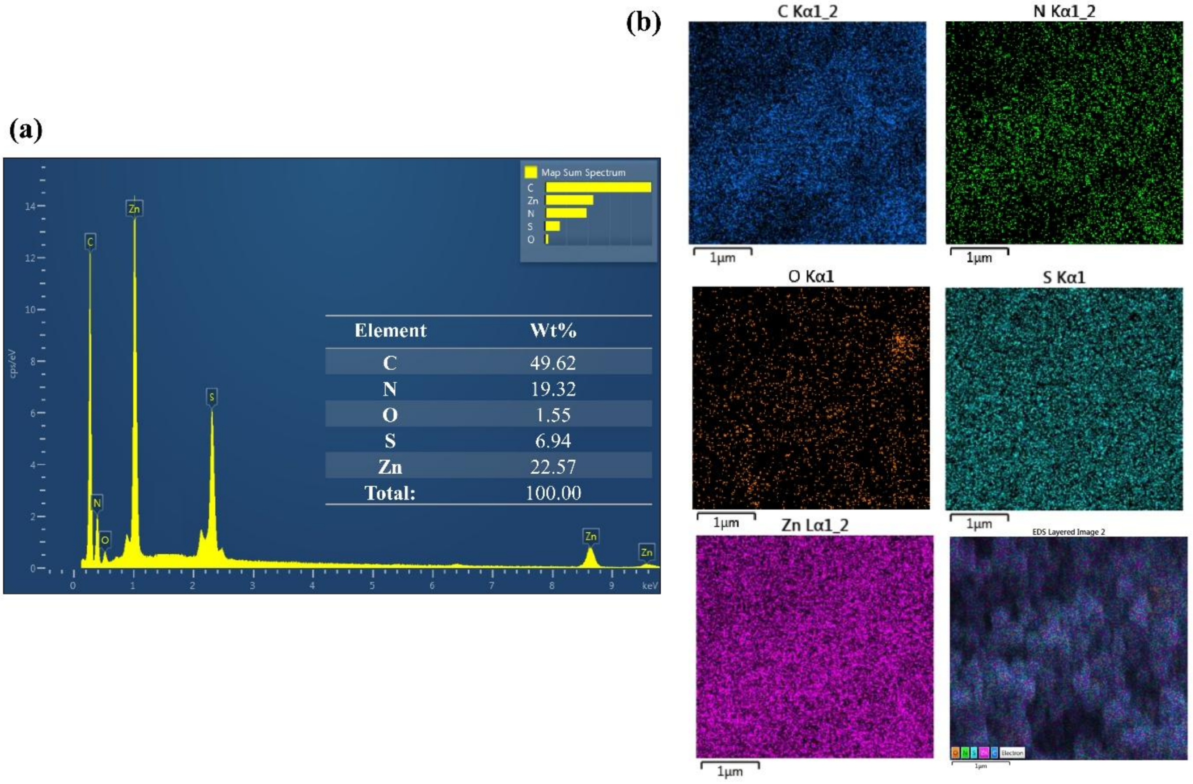

Fig. S8. (a) The EDAX spectrum and (b) elemental dot-mapping of the recycled ZIF-8 particles.

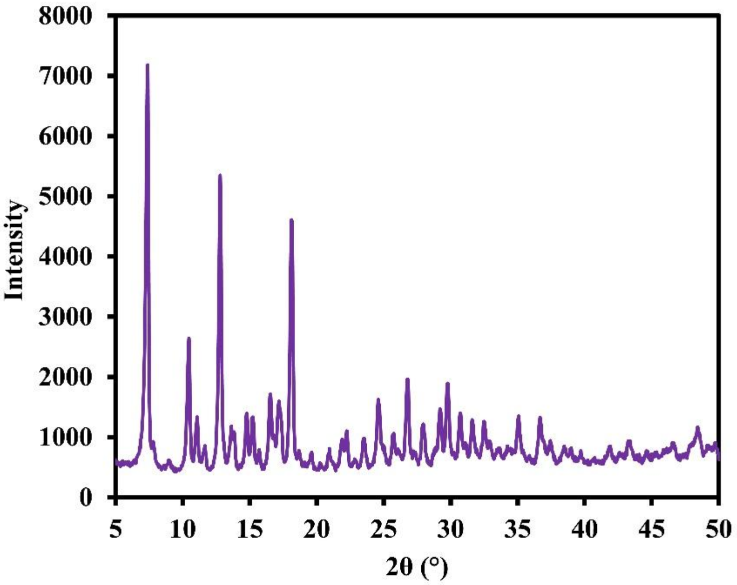

Fig. S9. The PXRD pattern of ZIF-8 after completing the oxidation reaction of toluene (180 minutes) at the pH of 5.

References

1. Y. Pan, Y. Liu, G. Zeng, L. Zhao and Z. Lai, *ChemComm* 2011, **47**, 2071-2073.
